# Dynamic Aspects of Pre-Soft Contact Lens Tear Film and Their Relation to Dry Eye: Basic Science and Clinical Relevance

**DOI:** 10.3390/life13040859

**Published:** 2023-03-23

**Authors:** Norihiko Yokoi, Petar Eftimov, Georgi As. Georgiev

**Affiliations:** 1 Department of Ophthalmology, Kyoto Prefectural University of Medicine, Kyoto 602-8566, Japan; 2 Department of Cell and Developmental Biology, Faculty of Biology, Sofia University “St. Kliment Ohridski”, 1164 Sofia, Bulgaria; 3 Department of Optics and Spectroscopy, Faculty of Physics, Sofia University “St. Kliment Ohridski”, 1164 Sofia, Bulgaria

**Keywords:** pre-lens tear film dynamics and stability, breakup patterns, soft contact lens, wettability, tear film lipid layer spread, dry eye

## Abstract

Soft contact lens (SCL) perturbs the intimate connection between the pre-lens tear film (PLTF) and the ocular surface in various ways, i.e., (i) decrease in tear meniscus radius and aqueous tear thickness, (ii) attenuation of tear film lipid layer spread, (iii) limited wettability of SCL surface, (iv) increased friction with eyelid wiper, etc. This often results in SCL-related dry eye (SCLRDE) manifested as PLTF instability and contact lens discomfort (CLD). In this review, the individual contributions of factors (i–iv) to PLTF breakup patterns (BUP) and CLD are considered via the tear film-oriented diagnosis framework adopted by the Asia Dry Eye Society from a clinical and basic science perspective. It is shown that SCLRDE (due to aqueous deficiency, increased evaporation, or decreased wettability) and BUP of PLTF classify within the same types as the ones observed for the precorneal tear film. The analysis of PLTF dynamics reveals that the inclusion of SCL enhances the manifestation of BUP associated with (i) decreased thickness of PLTF aqueous layer and (ii) limited SCL wettability as shown by the rapid expansion of BUP area. PLTF thinness and instability result in increased blink-related friction and lid wiper epitheliopathy as major contributor to CLD.

## 1. Introduction

When a soft contact lens (SCL) is inserted at the ocular surface, the intimate relationship between the precorneal tear film (PCTF) and the corneal surface epithelium is perturbed. This may result in contact lens discomfort defined by the Tear Film & Ocular Surface Society workshop as “a condition characterized by episodic or persistent adverse ocular sensations related to lens wear either with or without visual disturbance, resulting from reduced compatibility between contact lens and the ocular environment, which can lead to decreased wearing time and discontinuation” [1]. It was concluded that one of the most important premises to solve SCL discomfort is to clarify the mechanisms behind the instability of pre-lens tear film (PLTF), which is also important to understand the pathophysiology of SCL-related dry eye (SCLRDE) [2].

In a SCL wearer, both tear menisci and precorneal tear film (PCTF) are split into pre- and post-lens parts [3]. Then, at eye opening, the tears at the inferior pre-lens tear meniscus (PLTM) are pulled up by the eyelid to deposit the PLTF. Thus, by modification of the structure and radius of the PLTM, SCL presence alters the very process of TF deposition and formation. 

Once established, the PLTF often thins rapidly at 10–20 µm/min, far exceeding not only the PCTF thinning rates of 0.24–1.45 µm/min in healthy eyes but even the 7 µm/min observed for delipidated PCTF [4]. Therefore, it was long ago suggested that evaporation, although omnipresent at the ocular surface, cannot be the only factor behind PLTF dynamics. Other factor thought to be crucially important is the difference in the wettability and durability of SCL surface compared to the corneal surface [4,5]. In contrast to the cornea whose wettability is maintained by membrane-associated mucins, especially the longest MUC16, SCL surface has intrinsic limitations as the contact lens lacks such glycocalyx coating. Indeed, while rabbit cornea maintains complete wetting (i.e., zero contact angle upon deposition of sessile water droplets) for ninety minutes after enucleation [6], soft contact lens displays finite contact angles within a broad range of 10° to 70° (depending on the SCL material and the measurement techniques used) even immediately after removal from the blister pack [7]. SCL wettability in the course of wear may be further diminished (i) by the in vivo deposition of proteins or lipids to the contact lens or (ii) by the increased susceptibility to adverse environmental conditions (low temperature, low humidity, etc.) due to the thinner aqueous layer of the PLTF (as compared to the PCTF) [8]. In contrast to the corneal surface, SCL surface has no regenerative property; it might be deteriorated to less wettable depending on the wearing time, which also leads to the association with decreased wettability [9]. 

Therefore, although SCLRDE is often considered as evaporative dry eye (EDE) [10], such a view does not reflect the plethora of perturbations that SCL wear exerts at the ocular surface. The Asia Dry Eye Society classifies dry eye (DE) into three types, viz. aqueous deficient DE (ADDE), increased evaporation DE (IEDE), and decreased wettability DE (DWDE), which allows to encompass the diverse challenges imposed on the tear film by SCL wear [11,12,13,14]. Thus, from the classification point of view, it is a matter of great interest what types of DE are associated with SCLRDE within the framework developed by the Asia Dry Eye Society. 

Therefore, in this review, focusing on the pre-lens tear film dynamics, the mechanisms of the instability of PLTF and of SCLRDE are discussed from the perspective of basic science and clinical relevance. In the following sections, firstly (i) the clinical data on the effects of SCL wear on tear meniscus radius, tear film lipid layer (TFLL) spread, and PLTF breakup time and patterns will be presented (Section 2) and then, (ii) the theoretical perspective will be emphasized on how the SCL-induced perturbations of the ocular surface influence PLTF formation and stability (Section 3). 

## 2. Structural and Functional Change of Tear Film in Soft Contact Lens Wear

The major goal of our contribution is to demonstrate the application of tear film-oriented diagnosis (TFOD) and tear film breakup patterns (BUP) classification as performed by the Asia Dry Eye Society [11,12,13,14] to the analysis of PLTF dynamics and (in)stability. The implementation of TFOD and BUP classification require comprehensive information about the PLTF dynamics, i.e., simultaneous knowledge about the SCL-induced perturbations of tear meniscus radius, TFLL spread grade, PLTF breakup time, and breakup pattern (i.e., size, location, and degree of expansion of the dry spot). We illustrate the abovementioned clinical phenomena with data (Section 2.1 and Section 2.2) that were obtained by our team in the last decade [15,16,17,18]. The volunteers were healthy individuals without signs of dry eye or other ocular surface diseases, i.e., all the perturbations in PLTF dynamics can be unambiguously attributed to the insertion of SCL. These SCL-induced clinical phenomena at the ocular surface were reported also by other teams and key publications are referenced further. However, the data collected by our team are valuable considering the fact that the application of a broad range of mutually complimentary techniques allows to simultaneously and comprehensively evaluate how the different properties of PLTF change after SCL insertion and what happens prior to the fitting and shortly (30 min) after the removal of the SCL. Such observations are relatively rarely reported in the literature especially concerning the size, location, and degree and rate of expansion of breakup area(s), i.e., information important for the diagnostic algorithm of TFOD. Section 2.2 briefly summarizes the basics of the tear film-oriented diagnostics based on the analysis of tear film breakup patterns as developed in previous publications [11,12,13,14]. The relevance of TFOD to the analysis of PLTF dynamics is shown. Then, in Section 2.3 the importance of the increased friction realized between the SCL and the eyelid wiper due to the PLTF thinness and instability (i.e., PLTF provides less efficient lubrication than the “healthy” PCTF) is outlined. 

### 2.1. Alteration of TMR, TFLL Spread and PLTF Stability in SCL Wear

**Figure 1:**
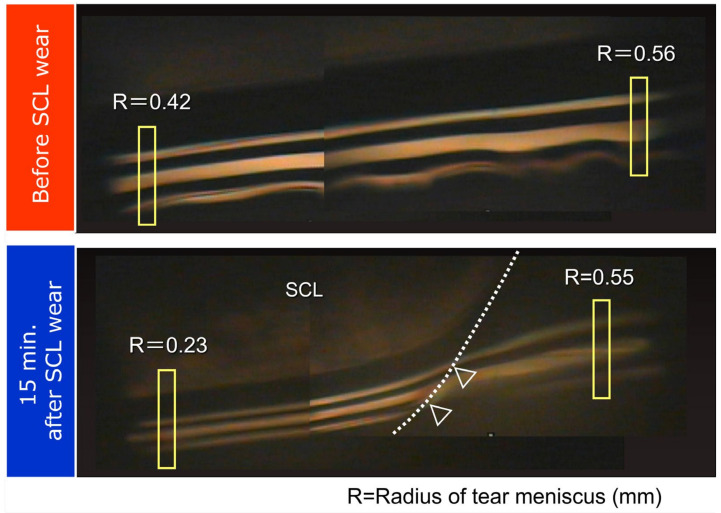
Tear meniscus change before and 15 min after SCL insertion. The upper image is the meniscometry image at the meniscus before SCL insertion, and lower image is that at 15 min after. Tear radius of tear meniscus (R) is attenuated only at the region where the SCL is inserted, which suggests that the meniscus tear volume is decreased only at the region. R: radius of tear meniscus (mm); SCL: soft contact lens. In the lower image the dashed line indicates imaginary margin of SCL behind the pre-SCL tear film and the arrow heads indicate the portion of the imaginary margin of SCL behind the pre-SCL tear meniscus.

In our SCL research, changes of tear volume, TFLL spread, and PLTF stability were evaluated noninvasively. Video-meniscometer [19,20] was employed for assessing tear meniscus radius (TMR). A video-interferometer, DR-1^TM^ (Kowa, Tokyo, Japan), was used [8] for the noninvasive evaluation of (i) the pattern and spread of TFLL (which both also qualitatively report about aqueous tear thickness [8] and (ii) the PLTF stability in terms of noninvasive breakup time (NIBUT) [8]. In our previous studies [15,16,17,18,21], it was found that after SCL insertion, (i) PLTM radius gets smaller than that of the original tear meniscus without SCL and (ii) that outside of the SCL, TMR is equivalent to the meniscus radius without SCL (Figure 1).

**Figure 2:**
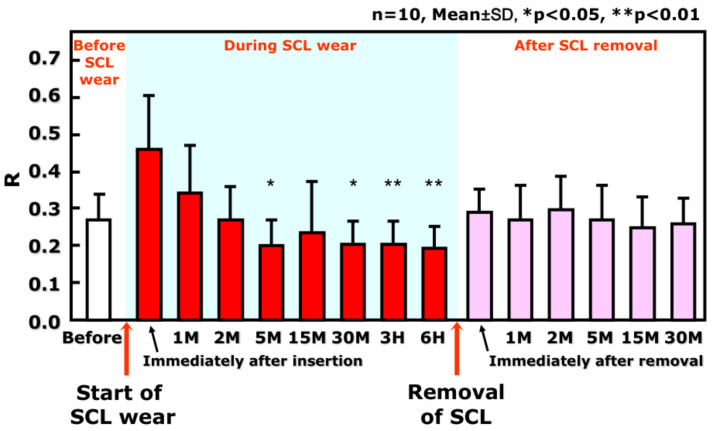
Time-Dependent Change in the tear meniscus radius (R), before, during, and after SCL wear R at the central lower tear meniscus corresponding to the meniscus tear volume, before, during, and after SCL wear. R is reduced as late as several minutes, say, 5 min after SCL insertion, maintained during wear, and is restored to that before SCL insertion immediately after SCL removal. R: radius of tear meniscus (mm); SCL: soft contact lens; M: minutes; H: hours. The figure is adapted with permission from reference [18] (Copyright year: 2015, copyright owner: The Japan Contact Lens Society).

**Figure 3:**
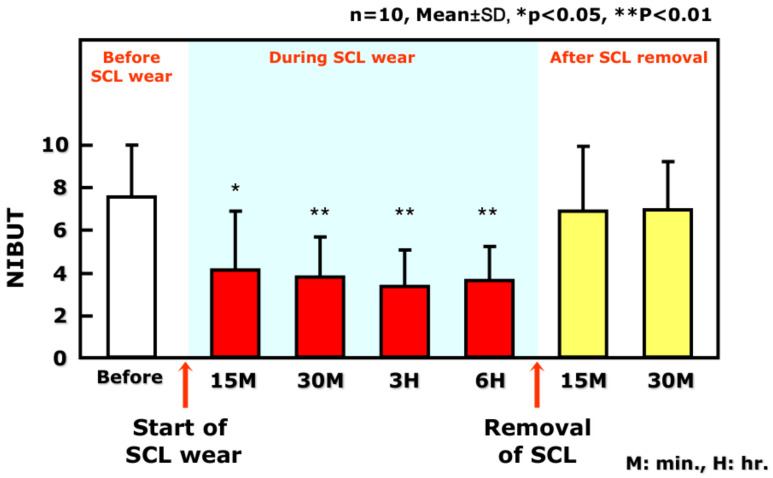
Time-Dependent Change in noninvasive breakup time (NIBUT), of the pre-lens tear film before, during, and after SCL wear. NIBUT corresponding to pre-LTF stability is reduced as late as 15 min after SCL insertion, is maintained during SCL wear, and is restored to that before SCL insertion immediately after SCL removal. SCL: soft contact lens; NIBUT: noninvasive breakup time (s); M: minutes; H: hours. The figure is adapted with permission from reference [18] (Copyright year: 2015, copyright owner: The Japan Contact Lens Society).

These results show that TMR, an indicator for the meniscus tear volume [20], is decreased at the area where SCL is inserted. After the removal of the SCL, the original TMR is restored to the value prior to the SCL insertion, indicating that lowered TMR is a transient change observed only during the SCL wear (Figure 2). A similar pattern is observed regarding noninvasive breakup time (NIBUT), i.e., SCL insertion results in a temporary decrease in PLTF stability, which rapidly recovers to normal when SCL is removed (Figure 3). 

**Figure 4:**
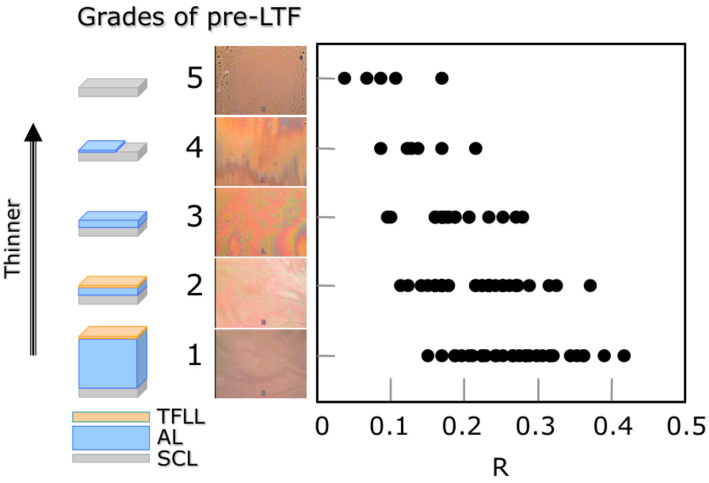
Relationship between R and interference grades of the pre-lens tear film. Positive correlation is seen between R and interference grades of pre-LTF (r_s_ = −0.597, *p* < 0.0001, n = 100), suggesting that an eye with lower tear volume is more susceptible to the thinner PLTF, which would lead to SCL-related dry eye. PLTF thickness is classified into one of 5 grades as shown here. In this classification, a greater grade corresponds to a thinner PLTF: Grade 1: Interference observed only from the tear film lipid layer (TFLL); Grade 2: Interference from the TFLL as well as a thin aqueous layer (AL); Grade 3: Interference from only a thin AL. TFLL is no longer present; Grade 4: Thin AL and the exposed surface of the SCL; Grade 5: No TF at all on the surface of the SCL. R: radius of tear meniscus (mm). One hundred eyes from 50 contact lens wearers were enrolled (60 eyes from 30 males; 40 eyes from 20 females; 32.2 (mean) ± 6.4 (SD) years old; with no dry eye) and they wore brand-new daily-use, one-day disposable soft contact lenses in the study. Measurement of radius of tear meniscus, which was followed by measurement of NIBUT, was performed 15 min after wearing of soft contact lens. The figure is adapted with permission from reference [18] (Copyright year: 2015, copyright owner: The Japan Contact Lens Society).

**Figure 5:**
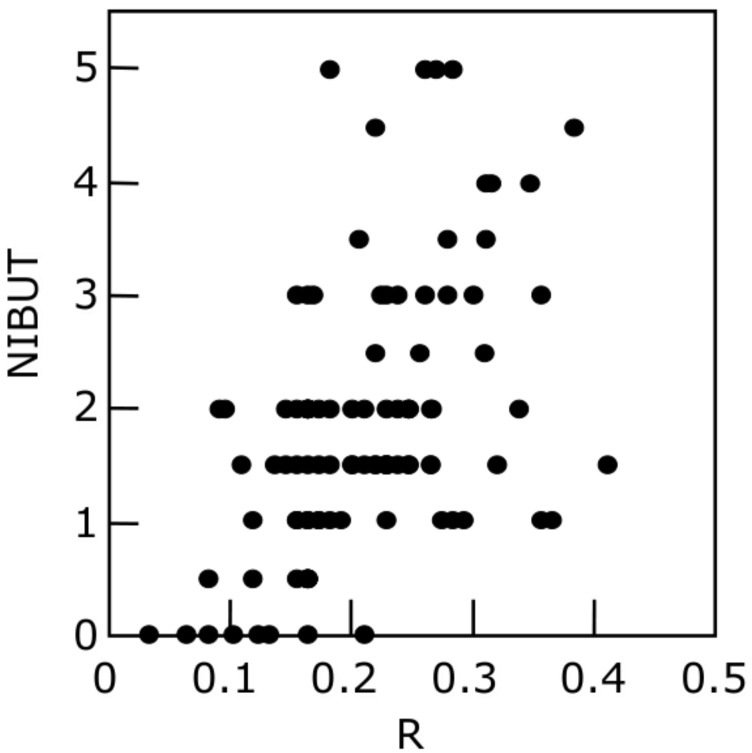
Relationship between R, the meniscus radius, and NIBUT. Positive correlation is seen between R and NIBUT (r_s_ = 0.476, *p* < 0.0001, n = 100), suggesting that an eye with lower tear volume is more susceptible to the instability of the pre-lens tear film which would lead to SCL-related dry eye. NIBUT: noninvasive breakup time (s); R: radius of tear meniscus (mm). One hundred eyes from 50 contact lens wearers were enrolled (60 eyes from 30 males; 40 eyes from 20 females; 32.2 (mean) ± 6.4 (SD) years old; with no dry eye) and they wore brand-new daily-use, one-day disposable soft contact lenses in the study. Measurement of radius of tear meniscus, which was followed by measurement of NIBUT, was performed 15 min after wearing of soft contact lens. The figure is adapted with permission from reference [18] (Copyright year: 2015, copyright owner: The Japan Contact Lens Society).

As demonstrated theoretically and clinically (see also Section 3) [22], there is a positive linear relationship between the radius of the lower tear meniscus and the aqueous layer thickness of PCTF [23,24,25]. Consequently, a similar relationship should be anticipated between the radius of the PLTM and the aqueous layer thickness of the PLTF. Hence, it is expected that in case of lower radius of the PLTM, PLTF thickness decreases, which in turn should manifest in (i) deteriorated grade of TFLL (Figure 4) and (ii) lower stability and shorter NIBUT of PLTF (Figure 5). All these phenomena were indeed observed clinically as shown in Figure 4 and Figure 5 and are foreseen theoretically (see Section 3). PLTM radius is recovered after the removal of the SCL (Figure 2); therefore, the less stable PLTF during SCL wear is also a transient phenomenon, suggesting that the CL-related discomfort and SCLRDE are also transient symptoms and transient complications, respectively. In this regard, for the prevention of SCLRDE, an increase in aqueous tear volume is of essential importance, as it will result in increased tear film thickness and hence a raised PLTF stability. 

The SCL-induced decrease in tear meniscus radius and/or volume with accompanying drop in the PLTF thickness and noninvasive breakup time of healthy individuals is reported in multiple studies [3,26,27,28] including in 84 young (22.4 ± 2.6 years) healthy volunteers (i.e., a group characterized with stable and robust tear film) [29]. Particularly interesting in the context of dynamic changes in TMR is the study of Chen et al. [30] showing continuous decrease in the lower tear meniscus volume of healthy individuals from 0.6 µL to 0.48 µL (i.e., drop of TMR from 0.3 to 0.2 mm [20]) along 2 to 10 h of continuous SCL wear, which is in very good agreement with the magnitude of the effect observed by us.

The impaired spread of TFLL due to the thin PLTF (it is generally thought that PLTF is 1.5 µm thinner than the PCTF of a healthy individual) is also reported by multiple studies with various observational techniques such as Lipiview, Tearscope, video interferometry, etc. [31,32,33,34]. Guillon proposed a grading scale of the TFLL images obtained by tearscope which confirmed that in presence of SCL, the spread of TFLL is suppressed and the lipid layer structure becomes patchier and more heterogeneous [33,35], i.e., a result identical to that shown at Figure 4. The advantage of DR-1 observations is that compared to Lipiview or Tearscope evaluation, the visualizations are of higher resolution, contain more details, and encompass the entire ocular surface which facilitated the unambiguous interpretation of the results obtained.

### 2.2. Breakup Pattern of PLTF in SCL Wear

**Figure 6:**
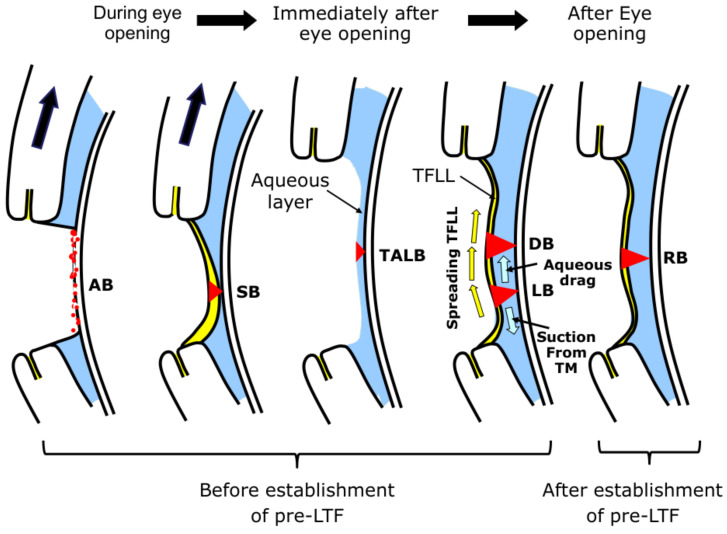
Classification of Breakup Patterns of PLTF with eye opening and with the eye kept open. Here yellow arrows imply upward spreading of TFLL and black arrows imply eyelid opening. See Section 2.2 and Section 3 of the main manuscript for more detailed explanation of the events involved in pre-lens tear film dynamics at eye opening and at interblink.

**Figure 7:**
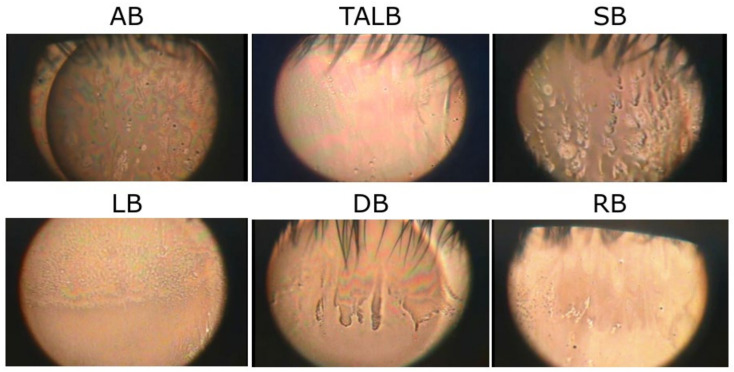
Classification of Breakup Patterns of Pre-LTF. AB: area break; TALB: thin aqueous layer break; SB: spot break; LB: line break; DB: dimple break; RB: random break.

DE is manifested by unstable TF [10,36] and according to the Asia Dry Eye Society classification, different types of DE are characterized by different breakup patterns (BUPs) of the tear film identified both via invasive (fluorescein staining) and noninvasive (DR-1^TM^ video-interferometer) observations of PCTF [12,14,37]. As will be discussed further (here and in Section 3), the noninvasively observed BUPs are in good agreement with the theoretical and clinical knowledge of the dynamic changes that take place in the PCTF upon eye opening and at interblink. As these processes remain relevant, when CL is inserted at the ocular surface it can also be expected that identical BUPs will be observed in PLTM as well. At the SCL surface, just like at the corneal one, the TF establishment is realized in two steps involving (i) deposition of aqueous tears to the SCL surface at the upward movement of the upper eyelid and (ii) subsequent redistribution of the aqueous tears and of the tear film lipid layer across the PLTF. The processes involved in the PLTF formation together with the possible resultant BUPs as observed by DR-1^TM^ are introduced below in agreement with the previously developed classification of tear film breakup patterns [12,14,37] (Figure 6 and Figure 7).

(1) Deposition of aqueous tears to the SCL surface occurs at eye opening when the capillary suction pressure [38] from the upper PLTM pulls the aqueous tears up from the lower PLTM, which results in the coating of aqueous tears at the SCL surface. The efficiency of the deposition depends both on the meniscus radius and on the wettability of the SCL [9]. Thus, if the volume of PLTM is extremely diminished, there is no efficient deposition of aqueous tears on the surface of the SCL at all. This BUP corresponds to the “Area break” typical for severe aqueous tear deficient dry eye [21,22,37]. In slightly less severe cases of PLTM reduction, a limited amount of AT is deposited at the SCL surface to yield a PLTF that is too thin and does not allow for lipid layer spread (and the associated upward drag of aqueous tears) to proceed over it [39]. In this case, within 1 s, Thin-Aqueous Layer break occurs [37], which is also associated with the rapid expansion of the BUP due to the rapid drying and diminished wettability of the SCL surface. Sometimes, although rarely, the so-called “Spot break” (analogous to the spot break BUP of PCTF) can be observed at eye opening when the wettability of the SCL is locally impaired after extended wear due to the excessive deposition of lipids or proteins over a discrete region of the SCL, which results in rapid local rupture of the PLTF over that region.
(2) After eye is opened, and if a sufficiently thick aqueous tear film is established, upward spread of the TFLL occurs analogously to the dynamics of PCTF formation. The leading edge of the lipid layer spread drags the underlying aqueous tears upwards, which results in transient thinning of the aqueous layer downwards and its thickening upwards [39,40,41]. During this redistribution process of PLTF, two distinct BUPs of PLTF are differentiated, “Line break” and “Dimple break”, which at this stage of tear film formation are also seen in PCTF, i.e., in dry eye over the native (in absence of SCL) corneal surface [12,14,37]. However, it should be noted that as the SCL surface is inherently less wettable than the corneal surface, both “Line break” and “Dimple break” of PLTF are accompanied by rapid expansion of the breakup area [12,14]. If PLTF remains stable until completion of the upward spread of the TFLL (i.e., in cases of higher number of aqueous tears at TMR ≥ 0.3 mm), after the cessation of TFLL upward spread, breakup appears as “Random break” corresponding to the eponymous BUP of the PCTF in DE without SCL wear. Random break may be associated with rapid expansion of the dry (i.e., breakup) area when the wettability of SCL surface is impaired.

Among those BUPs seen at the SCL surface, it may be suggested that “Area break” and “Thin aqueous layer break” are associated with relatively severe aqueous tear deficiency; “Spot break” is associated with the deterioration of the SCL surface; and Line break and Dimple break both with rapid expansion of the dry area are associated with decreased wettability of SCL surface. Random break occurs in cases with higher aqueous tear volume when thicker PLTF is formed. However, further investigation is necessary to clarify those BUPs. The importance of BUP dynamics as a tool to evaluate PLTF interactions with SCL and for differential diagnosis of dry eye is recently increasingly recognized as an important diagnostic modality in (video)keratography studies where there is ongoing research for the implementation of TFOD [42,43].

### 2.3. Comprehensive Understanding of the Vicious Cycle of SCLRDE

**Figure 8:**
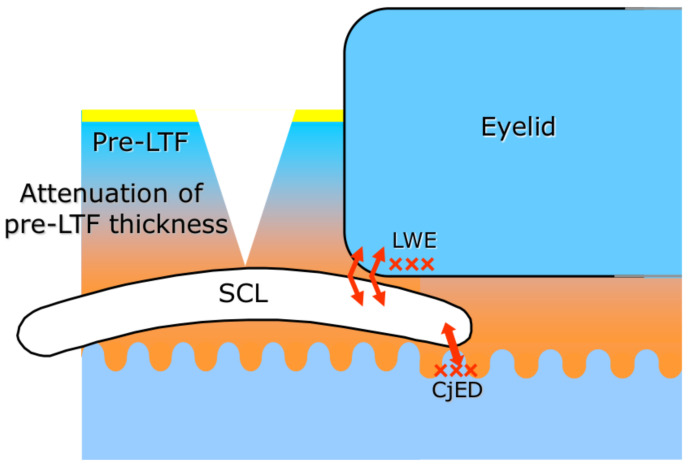
Possible mechanism for soft contact lens-related dry eye. When the lens is in place, the PLTF gets thin and unstable. As a result, blink-related friction occurs, leading to lid wiper epitheliopathy (LWE) and conjunctival epithelial damage (CjED), which in turn can cause contact lens discomfort (CLD). Here “x” implies the epithelial damage of the lid wiper portion or conjunctival epithelium and the arrows indicate the region of increased friction. In cases of aqueous tear deficiency, these mechanisms are more pronounced and the related CLD can be more severe.

Due to the transient SCL-induced decrease in TMR, the PLTF is significantly thinned similarly to the condition observed in aqueous tear deficient PCTF. As SCL protects the cornea, the decreased PLTF stability should not result in corneal desiccation. However, contact lenses in general have a higher coefficient of friction than the human cornea, which deteriorates further with SCL dehydration at wear [44,45]. Therefore, the lack of thick and stable TF over the SCL should result in increased (higher than the one in precorneal tear film (PCTF)) blink-related friction and this mechanism should play a major role in the symptoms of contact lens-related discomfort and eye pain in SCLRDE (Figure 8).

Indeed, as described in an earlier review on the application of TFOD to SCL-related dry eye [46], lid wiper epitheliopathy (LWE) was found in 80% of SCLRDE subjects but in only 13% of asymptomatic contact lens wearers and thus an association is revealed between CLRDE and LWE [47]. In Japan, Shiraishi et al. [48] also reported a high frequency of LWE among contact lens wearers. A number of recent reviews highlighted LWE as a major factor associated with SCL dropout as well [49,50].

This was also shown in our previous study [18], where the radius of the PLTM decreases compared with the original PCTM within 15 min after SCL insertion (Figure 2), which results in thinner PLTF. This in turn leads to the increased friction between the SCL surface and the lid wiper portion (with associated epithelial damage at the lid wiper portion) [47] and between the edge of the SCL and the conjunctival surface [51]. Furthermore, eye dryness during SCL wear is found to be related to tear volume, conjunctival staining, and lid wiper epitheliopathy (LWE) scores [18,21]. However, no significant relationship was noted between corneal epithelial damage and eye dryness. Therefore, the SCL-covered corneal surface is protected against both desiccation and increased friction (as there is no direct contact with the lid wiper). Thus, the increased blink-related friction due to the thinned PLTF causes contact lens discomfort via two other mechanisms: (i) lid wiper epitheliopathy and (ii) conjunctival epithelial damage induced by the lens edge. It was found [15,16,17,18] that more than 3 h are needed for CLD to occur despite very early decrease in the PLTM radius and the attenuation of PLTF noninvasive breakup time only 30 min. after SCL insertion. Thus, commonly observed were cases with extreme dryness during SCL wear in which, despite minimal or no corneal epithelial staining after SCL removal, severe LWE and bulbar conjunctival damage were displayed. These results are in excellent agreement with previous reports that the severity of LWE [47] and the severity of bulbar conjunctival damage are associated with the severity of contact lens discomfort (CLD) [51]. Therefore, in SCL wear, it is not exaggerated to say that the problem of corneal surface damage due to the instability of PCTF is shifted to that of conjunctival surface damage because of increased blink-related friction due to the instability of PLTF. 

Recently, greater attention has been paid from the point of view of material science to the enhancement of wettability and lubricity at the stage of SCL materials design [44]. To overcome SCLRDE and resultant CLD, SCLs with enhanced wettability and lubricity, and eye drops which can increase the PLTM thickness and/or decrease the friction, can be suggested as beneficial and further development has been advancing.

## 3. Some Fundamental Aspects of PLTF Dynamics

Already in 2005, Nichols et al. [4] mentioned that although SCLRDE is typically classified as evaporative dry eye due to the accelerated PLTF thinning rate, this is an oversimplification as CL presence may exert a variety of perturbations at the ocular surface. In line with these thoughts, the current study shows that CL may reduce tear meniscus radius, inhibit TFLL spread, or cause rapid PLTF breakup that might be more related to the CL wettability rather than to evaporation or to aqueous tear (AT) deficiency. All these factors, which were indeed found to take place at the ocular surface, are expected to reduce the stability of PLTF in a specific manner distinct from the evaporation and will be discussed in the next subsections.

### 3.1. Pre-lens Tear Meniscus Radius, Aqueous Tear Film Thickness, and TFLL Spread

In the initial step of tear film formation, the upper eyelid deposits the aqueous tear across the ocular surface at upstroke similarly to the painting of a wall with a brush. The deposition efficiency is determined by (i) the tear meniscus radius (*R*, mm) and surface tension (*γ* = 43 mN/m) which set the capillary action of the meniscus, (ii) the eyelid velocity (*U*, cm/s) in the course of the upstroke, and (iii) the AT viscosity (*µ* = 0.013 cP) responsible for the viscous drag opposing to the deposition [52,53]. Furthermore, *R* determines the tear meniscus volume, which is a measure for the overall amount of AT present at the ocular surface [20]. 

Assuming a perfectly extensible lipid layer, the relationship between these parameters and the initial thickness (*h*) of the TF at deposition is given by Equation (1) [22]:

(1) $$h=1.338R{\left(µ\frac{U}{\gamma }\right)}^{2/3}$$

**Figure 9:**
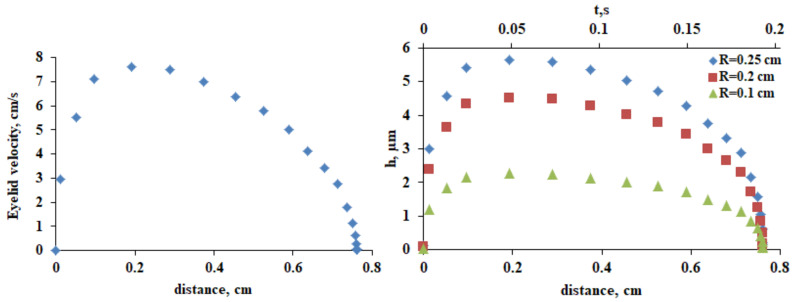
**Left panel**: The velocity of upper eyelid across the ocular surface. The eyelid traverses the distance for 0.2 s. **Right panel**: Thickness profile of PLTF immediately after deposition as calculated by Equation (1). At both panels, *x* = 0 is positioned at the lower eyelid margin.

The spatiotemporal eyelid velocity distribution [52,53] is known from the literature (Figure 9, left panel) and the upper and lower tear meniscus radius and the CL influence on them are known to be similar [54]. Thus, it is possible to quantitatively evaluate the impact of the reduction of tear meniscus radius caused by CL insertion as found here and in previous studies (Figure 9, right panel).

It should be kept in mind that the PCTF thickness profile calculated with Equation (1) represents *h* values instantaneously at deposition, i.e., prior TFLL upward spread and black line formation to take place, which results in 1–1.5 µm thinning of TF over the central cornea. Thus, these thickness profiles are somewhat different from the commonly measured stationary shapes reported in literature. Still, the calculated TF thicknesses are well within the range of TF thickness values measured with high resolution optical coherence tomography (OCT) [55].

As can be seen, the decrease in tear meniscus radius typical in the presence of CL reduces the thickness of the AT deposited at eye opening, similarly to the complications arising in the case of ADDE. The theoretical result is in excellent agreement with the clinical data here as well as with previous studies on SCL-related dry eye [56,57]. It was reported that CL wear reduces by one-third the tear meniscus volume, which is associated with a decrease in the total tear volume and of the AT thickness, which in turn correlates with the degree of CL discomfort and with accelerated destabilization of the PLTF [42,58].

The significantly decreased thickness of the AT layer upon deposition not only facilitates rapid breakup by its own self but it also prevents the next crucial step of the TF formation: the upward spread of TFLL from the lower to the upper tear meniscus [59,60,61,62]. This is so because in order for TFLL spread to take place, a sufficiently thick AT layer is obligatory in order to prevent friction of TFLL with the CL surface [39]. In turn, TFLL spread drags the underlying AT upwards, which results in redistribution of the AT (i.e., thinning of the TF at the lower and central part of the ocular surface and TF thickening at the upper cornea) to more uniform TF thickness profile across the ocular surface [39,40,41].

The rheology of TFLL spread is well described by the Voigt law (Equations (2) and (3)) [59]:

(2) $$H\left(t\right)=H_{max}[1-\exp(-\frac{t}{\tau })],$$

(3) $$H_{max}=V_{0}\tau$$

Here, *H* is the distance (the height) travelled by the TFLL at time *t*, *H_max_* is the maximum height reached when TFLL spread is complete, *V*_0_ is the initial TFLL velocity at *t* = 0, *τ* is characteristic time defined by the ratio of TFLL elasticity to the friction experienced by TFLL upon spread, and t is time.

Analysis of clinical data revealed strong and statistically significant correlations between the rheological and the clinical parameters, which revealed that with the increase in AT thickness (and of tear meniscus radius, respectively) the velocity, the characteristic spread time, and the maximum height travelled by the TFLL are enhanced: *V*_0_ ≈ 0.43718 × *h*^2^ (R = 0.75; *p* = 0.017) and *τ* ≈ 1.7211 × *h*^−1^ (R = 0.64; *p* = 0.014) (in these empirical correlations, *t* is in seconds, *V*_0_ in mm/s, and *h* in micrometers) [59,60,61,62].

**Figure 10:**
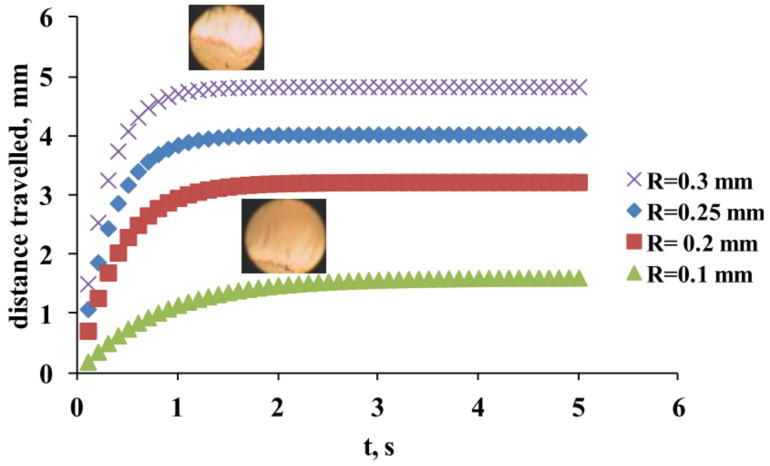
Higher tear meniscus radius means higher thickness of AT layer at deposition and faster and better spread of TFLL (Equations (2) and (3)), as illustrated by the image inserts. The calculated curves are in very good agreement with the trends of clinical data.

This semiempirical framework allows to elucidate the typical impact of the decrease in *R* (i.e., of AT thickness) on the TFLL spread (Figure 10).

As can be seen, the reduction in tear meniscus radius (i.e., of AT volume across the ocular surface) results in slower and incomplete TFLL spread, which in turn prevents the formation of proper tear film [59]. These estimates are in very good agreement with the clinical results reported previously [59,60,61,62]. Thus it can be seen that the SCLRDE in which tear meniscus radius is decreased is more similar in its mechanism to ADDE, rather than to evaporative dry eye [14]. In addition, the CL-related reduction of tear meniscus radius is strongly correlated with certain breakup patterns (primarily “Area break”, “Thin aqueous layer break”, and “Line break”) which are typical for severe and mild to moderate aqueous tear deficiency, respectively, when precorneal tear film instability is concerned [14]. An important difference between these forms of SCLRDE and typical ADDE is that in the case of SCLRDE, the reduction of tear meniscus radius and of AT thickness at deposition is often not due to the inherent AT deficiency due to lacrimal gland dysfunction but due to the perturbation of tear meniscus structure by the contact lens. Thus, once the SCL is removed from the eye, the tear meniscus curvature is restored to normal and the stability of the precorneal tear film returns to normal as well [42,58]. 

### 3.2. CLs and TF Thinning Rate in the Region of the Blackline

Another very important phenomenon in healthy and “dry” eyes related to the capillary action of tear menisci is the formation of “black line”, i.e., dark (when visualized with fluorescein) thin (≤200 nm) lines near the upper and lower lid margins, immediately following a blink [38] and resulting in the formation of a “perched tear film” over the central cornea. Although TF may not break up at black lines, the faster their formation is, the higher is the suction of aqueous tears towards the tear menisci and the faster the thinning of the adjacent TF regions. This in turn promotes TF instability starting over the lower towards the central cornea [63].

Numerical solutions of the thin film equation allowed Sharma et al., 1998 [64] to derive a hydrodynamic model (Equation (4)) for the meniscus-induced thinning of the tear film:

(4) $$t=3.52\;{\left(\frac{R}{h_{m}}\right)}^{2.232}{\left(\frac{h_{0}}{R}\right)}^{1.268}\left(µ\frac{RC}{\gamma }\right)$$

Here, *t* is TF thinning time, *R* is tear meniscus radius, *h*_0_ and *h_m_* are the initial and minimal TF thickness, respectively, *µ* is tear viscosity, and *γ* is tear surface tension. Equation (4) also contains the term *C* which accounts for the capability of the TFLL to provide tangentially immobile air/tear surface (Gibbs-Marangoni effect). *C* = 1 for a “free” tear surface of zero shear stress, whereas *C* = 4 when the tear film surface is immobilized by the lipid layer. A very similar solution of the thin film equation for the meniscus-induced TF thinning was also derived by Wong et al., 1996 [52]. 

**Figure 11:**
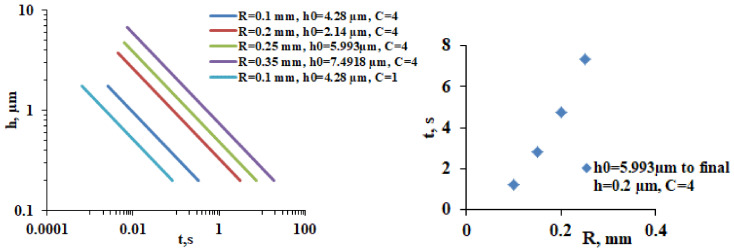
**Left panel**: lower tear meniscus radius is associated with lower AT thickness and faster thinning as calculated by Equation (4). The final thickness at the black line is 0.2 µm. **Right panel**: Illustration of the “thirsty meniscus phenomenon”: at equal other conditions, lower tear meniscus radius has stronger suction capillary pressure and greatly accelerates film thinning.

Equation (4) allows to model the impact of CL-induced decrease in tear meniscus radius on the rate of meniscus-induced thinning (Figure 11).

As can be seen in Figure 11, left panel, the decrease in tear meniscus radius and the associated decrease in PLTF thickness result in faster thinning rates. The effect can be additionally emphasized if dysfunctional TFLL (*C* = 1) is included, a condition that is well possible in the case of a very thin AT layer (i.e., very low *R*), where TFLL spread is suppressed. The effect of tear meniscus radius at equal other conditions, i.e., equal AT thickness and TFLL spread (which is of course physiologically unrealistic) is simulated at Figure 11, right panel. It shows that the lower is the tear meniscus radius, the faster is the PLTF thinning. This is a demonstration of the so-called “thirsty” meniscus, i.e., lower *R* means higher tear meniscus curvature and stronger suction pressure exerted by the meniscus at the adjacent tear film. Such a trend is confirmed in clinical observation and is known to result in accelerated breakup of the TF at the lower cornea in ADDE [63]. Such behavior corresponds to the “Area break” and “Thin aqueous layer break” BUPs commonly observed over CL surface at low *R* values. Once again, this mechanism of accelerated instability of PLTF is related not with evaporation but with the higher suction pressure and AT deficiency at deposition induced by the more curved tear meniscus.

### 3.3. How CL Wettability May Alter PLTF Thinning Rate and Stability Away from the Black Line Region

In vivo studies revealed that even if successfully deposited (i.e., the CL-induced reduction of *R* is moderate), PLTF often has an abnormally high thinning rate of 10–20 µm/min compared to the “standard” PCTF thinning rates of 0.24–1.45 µm/min and even faster than the 7 µm/min observed for delipidated PCTF [4,65,66,67]. Such abnormally accelerated thinning cannot be explained solely by evaporation and is commonly attributed to dewetting [68]. This indeed can be expected, as most CLs display significant water contact angles (θ = 30° to ≥60°) immediately after removal from blister which tend to increase during wear [7,9]. In contrast, freshly enucleated rabbit cornea with intact glycocalyx displayed ideal wettability (θ = 0°) for 90 min after exposure to air [6]. 

In the eye, PLTF is continuously subjected to perturbations due to eyelid and eye movements, invasion by dust/cosmetic particles, and formation of hydrophobic nonwetting spots at the SiHy surface (due to silicone migration, lipid deposition, attachment of tear micro bubbles or other), etc. [64]. These perturbations may open transient holes of micrometric size in it, which might either (i) enclose, i.e., the PLTF is repaired and remains stable, or (ii) expand and result in dewetting of the SiHy and destabilization of the PLTF. Sharma and Ruckenstein [69,70] derived the relationship between the PLTF thickness and stability, the hole radius, and the water contact angle (i.e., the wettability) of the SiHy (Equation (5)):

(5) $$\left(\frac{h_{c}}{r}\right)=tan\;\theta \left[\sqrt{1+cos\theta }-1\right]\;,\;for\;\theta \leq \frac{\pi }{2}\;$$

Here, *h_c_* is the critical thickness below which the wetting film is no longer unconditionally stable and becomes susceptible to rupture if a hole, a dry spot, with radius r, is formed on the SiHy surface. For CA ≤ 45°, the *h_c_/r* ratio is <0.4, i.e., even thin (compared to the size of the defect) PLTF should remain stable. This means that for a nonwetting spot of 3–4 micrometer radius (as typically observed at the early stages of SiHy desiccation damage) [71], for any aqueous film thickness ≥1.6 µm, the wetting film will remain unconditionally stable. With the raise of *h_c_/r* due to macroscopic increase of the contact angle or due to locally compromised CL surface (i.e., due to lipid deposition, microbubble attachment, etc.), the situation changes. For an *h_c_/r* ratio of ≥0.5, the PLTF will become susceptible to rupture for *h* < 2 µm. This means that at the typical physiological PLTF thickness of 2–2.5 μm, dewetting becomes energetically favorable, which agrees very well with the rapid thinning and instability of PLTF commonly observed in the current study and elsewhere [39,40,41]. 

A critical moment in the TF formation that can promote the contamination of the CL surface with lipids/particles is the TFLL upward spread in which a Marangoni ridge is formed, i.e., thick AT is dragged upwards by lipid layer while AT thickness behind the advancing front is temporarily depressed which may result in TF destabilization over that region [39,40,41]. This redistribution of tear fluid is registered as up to 1.5 µm AT thinning over the central cornea within 1 s after eye opening [39,40,41]. This mechanism is thought to be operative for the line break and dimple break registered within 1–2 s at the CL regions located over the lower or central part of the cornea [12]. 

Apart from facilitating TF rupture at micrometric thicknesses, higher contact angle of the contact lens is also responsible for higher rate and degree of expansion of the dry area over the CL surface, which additionally enhances PLTF instability with the accumulation of desiccation stress during daily wear. A detailed review of the possible relationship between the rate and degree of expansion of dry spots and the biomaterial contact angles can be found in Bertand et al., 2010 [72]. 

The agreement between the basic science considerations of CL wettability and the in vivo observations of PLTF (in)stability patterns in the current study align with the clinical correlations between higher CL wettability (lower water contact angle) and higher comfort score and PLTF breakup times for a broad range of CL materials [73,74,75,76,77].

### 3.4. The Random Break Pattern

When all the steps of PLTF formation are completed and uniform tear film is established at the SCL surface, tear film breakup occurs at a random position within 5–10 s over the CLs. This type of breakup is promoted by evaporation, alone or in combination with other factors discussed above, and resembles the random break observed in healthy eyes without CL. 

Overall, it can be seen that the fitting of CL perturbs the ocular surface in multiple ways: reduction of the tear meniscus radius, inhibition of TFLL spread, and substitution of the highly wettable cornea with CL surface of limited wettability. All these perturbations may destabilize the PLTF in a unique way distinct from evaporation. This is indeed confirmed by the diverse type of PLTF breakup patterns that matched very well the PCTF instability patterns defined in the tear film-oriented diagnosis of dry eye developed for PCTF treatment [14,37]. 

## 4. Conclusions

In conclusion, this review discussed the mechanism of SCLDE from a clinical and basic science point of view. This scientific field needs interaction among clinicians, basic scientists, and SCL makers, and as far as SCL is important for the correction of vision, further advances are required to improve the safety of SCL to OS and decrease CLD.

## Data Availability

The data that support the findings of this study are available from the corresponding author, N.Y., upon reasonable request and also in the references quoted in the text.
